# An Acoustic Analysis of the Genus *Microhyla* (Anura: Microhylidae) of Sri Lanka

**DOI:** 10.1371/journal.pone.0159003

**Published:** 2016-07-12

**Authors:** Nayana Wijayathilaka, Madhava Meegaskumbura

**Affiliations:** Department of Molecular Biology and Biotechnology, Faculty of Science and Postgraduate Institute of Science, University of Peradeniya, Peradeniya, KY, 20400, Sri Lanka; University of Pavia, ITALY

## Abstract

Vocalizing behavior of frogs and toads, once quantified, is useful for systematics, rapid species identification, behavioral experimentation and conservation monitoring. But yet, for many lineages vocalizations remain unknown or poorly quantified, especially in diversity rich tropical regions. Here we provide a quantitative acoustical analysis for all four Sri Lankan congeners of the genus *Microhyla*. Three of these species are endemic to the island, but *Microhyla ornata* is regionally widespread. Two of these endemics, *M*. *karunaratnei* (Critically Endangered) and *M*. *zeylanica* (Endangered), are highly threatened montane isolates; the other, *M*. *mihintalei*, is relatively common across the dry lowlands. We recorded and analyzed 100 advertisement calls from five calling males for each species, except for *M*. *zeylanica*, which only had 53 calls from three males suitable for analyses. All four species call in choruses and their vocal repertoires are simple compared to most frogs. Their calls contain multiple pulses and no frequency modulation. We quantified eight call characters. Call duration and number of pulses were higher for the two montane isolates (inhabiting cooler habitats at higher altitudes) compared to their lowland congeners. *Microhyla zeylanica* has the longest call duration (of 1.8 ± 0.12 s) and the highest number of pulses (of 61–92 pulses). The smallest of the species, *Microhyla karunaratnei* (16.2–18.3 mm), has the highest mean dominant frequency (3.3 ± 0.14 kHz) and pulse rate (77 ± 5.8 pulses per second). The calls separate well in the Principal Component space: PC1 axis is mostly explained by the number of pulses per call and call duration; PC2 is mostly explained by the pulse rate. A canonical means plot of a Discriminant Function analysis shows non-overlapping 95% confidence ellipses. This suggests that some call parameters can be used to distinguish these species effectively. We provide detailed descriptions for eight call properties and compare these with congeners for which data is available. This work provides a foundation for comparative bioacoustic analyses and species monitoring while facilitating the systematics of *Microhyla* across its range.

## Introduction

Among amphibians, anurans (frogs and toads) are conspicuous for their calling (vocalizing) behavior [1–4]. Acoustically well-studied vocal repertoires of anurans provide vital information on many fronts: ascertaining species identities, especially when cryptic species are involved [5–11]; acoustic experimenting and understanding specific acoustic signaling in different behavioral contexts [12–15]; bioacoustic monitoring, surveys, remote sensing and population studies [16–18]. However, for this, the vocal repertoires of frogs need to be understood statistically. Such bioacoustic analyses for tropical regions of the world, where most of the anuran diversity exists, are almost non-existent [19,20].

This is also true for the genus *Microhyla*, widespread throughout the tropics across India, Sri Lanka, Indonesia, archipelagos of Ryukyu of Japan and Sulu of Philippines [21]. So far, 39 species of the genus have been identified [10,21], of which four (ca. 10%) are found in Sri Lanka. The island is now a well-recognized hotspot of amphibian diversity and endemicity [9,22,23], containing a unique assemblage of fauna and flora, distinct from Indian mainland, with only a few shared species between the two regions. The genus *Microhyla* also shows the same pattern of distribution, with three species being endemic to the island, and one shared species [10].

Due to their widespread nature, only 4 of the 39 species are listed as threatened by the IUCN, 2015 [24]; however, of these, two species, *M*. *karunaratnei* (Critically Endangered) and *M*. *zeylanica* (Endangered), are confined to two mountain peaks of the Central Hills and Rakwana Hills of Sri Lanka. Furthermore, *M*. *karunaratnei* is considered an EDGE (Evolutionary Distinct and Globally Endangered) species [25], the only species amongst *Microhyla* and the only Sri Lankan amphibian to be included in this category. Though these species are prioritized for conservation, their ecology, behavior and natural history is virtually unknown. A deeper understanding of these threatened species will enable their effective conservation.

The vocalizations of only a few species of *Microhyla* have been analyzed or published up to now. These include, *M*. *bornensis* (Borneo), *M*. *berdmorei* (Thailand), *M*. *butleri* (Thailand), *M*. *fissipes* (Thailand), *M*. *heymonsi* (Thailand, India), *M*. *laterite* (India), *M*. *petrigina* (Borneo), *M*. *ornata* (India) and *M*. *rubra* (India), *M*. *sholigari* (India) [26–33]. So far call characterization work has not focused on the Sri Lankan members of this genus except for a population of *M*. *ornata* from India [30] and *M*. *mihintalei*, a recently described species [10].

Here, we provide a quantitative description of the vocalizations for the three Sri Lankan endemic species, *M*. *karunaratnei*, *M*. *zeylanica*, *M*. *mihintalei*, and a Sri Lankan population of *M*. *ornata*. We provide descriptions of eight call properties measured for 353 calls from 18 individuals, which also help us evaluate the intraspecific variation within these species. We discuss and compare the vocalizations of these species and point out the knowledge gaps, thus providing the basis for species monitoring and systematics of *Microhyla* across its range.

## Materials and Methods

### Ethics Statement

Research was conducted under the permission of Department of Wildlife Conservation (permit no. WL/3/2/13/13) and Forest Department (permit no. R&E/RES/NFSRC/14) of Sri Lanka. Specific methods of collection, euthanasia, tissue sampling and fixation followed the guidelines for use of live amphibians and reptiles in field research by the American Society of Ichthyologists and Herpetologists (ASIH) (http://www.asih.org/pubs/herpcoll.html; dated 13 March 2006), and were approved by the ethical committee of Postgraduate Institute of Science, University of Peradeniya at its 16^th^ meeting held on 14^th^ November 2014.

### Fieldwork and acoustic recordings

**Fig 1:**
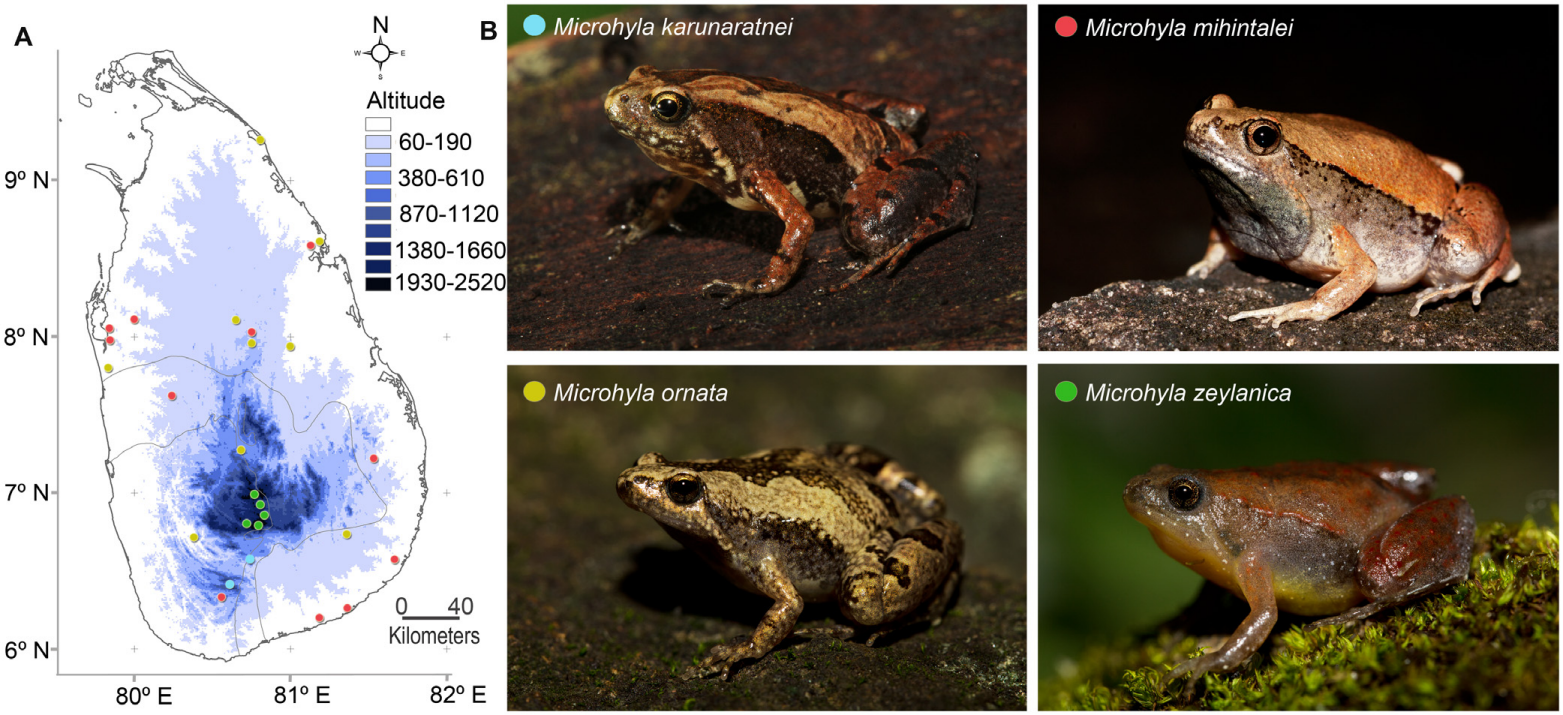
Four *Microhyla* species in life and their distribution. (A) A map showing the distribution of *Microhyla karunaratnei* (blue), *M*. *zeylanica* (green), *M*. *mihintalei* (red) and *M*. *ornata* (yellow). (B) Dorsolateral view of the four species (in life).

Calling males of *Microhyla karunaratnei* were recorded between 20:00–23:00 hours on 9^th^ December 2014 from a population in Morningside forest reserve, Suriyakanda, Rathnapura district (6.4075°N, 80.6094°E, 1050 m.a.s.l). They were calling around a breeding pool, an abandoned gem-pit in a regenerating forest patch with tall grasses and shrubs. Fairly common, calls of *M*. *mihintalei* and *M*. *ornata* were recorded from temporary breeding pools in Mihintale, Anuradhapura district (8.3548°N, 80.5054°E, 120 m.a.s.l) on 27^th^ September 2014 and Maakandura, Kurunegala district (7.3245°N, 79.9887°E, 30 m.a.s.l) on 25^th^ August 2014 respectively. Calls of *M*. *zeylanica* were recorded from a shallow ephemeral pool in a grassland habitat at Horton Plains National Park (6.7963°N, 80.8179°E, 2135 m.a.s.l) on 22^nd^ and 23^rd^ August, 2014 between 21:00–02:00 hours (Fig 1). Samples of call recordings used in the analyses with collection numbers of each species are provided as supporting information (S1–S4 Audio), which will help direct and wider comparison with other taxa [34].

Calls of five males from each species (except *M*. *zeylanica*, *N* = 3) were recorded using a digital recorder, Marantz PMD 620 MKII (sampling rate 44.1 kHz, 16-bit resolution) and a directional Sennheiser-ME66 microphone equipped with a foam windscreen. Microphone was handheld to maintain a distance of 0.3 to 0.5 m between calling male’s snout and the microphone tip. Gain setting of the recorder was adjusted prior to each recording and maintained until the end of the given recording. Ambient temperature of the calling site was taken immediately after each recording using a handheld Kintrex IRT0421 non-contact infrared thermometer to the nearest 0.1°C. Snout vent length (SVL) and body weight (BW) of all recorded males were measured *in situ* using a precision digital caliper and a portable digital balance to the nearest 0.01 mm and 0.01 g respectively; one specimen from each species were taken as a reference sample and all other animals were released back to their original habitat upon taking measurements. Collected male was placed in a moist plastic container (200 ml) with some grass blades or leaf litter and transported to the field station in less than 3 hours. It was then euthanized using Tricaine Methanesulfonate (MS-222), fixed in 4% formalin and preserved in 70% ethanol (*M*. *karunaratnei* DZ1530, *M*. *zeylanica* DZ1420, *M*. *mihintalei* DZ1445, *M*. *ornata* DZ1427). The species identification was done using morphology [10, 35–38]. Tissue samples (thigh muscles or liver) were taken immediately after euthanization and stored in absolute ethanol at -20°C for further analyses at the Department of Molecular Biology and Biotechnology, University of Peradeniya (reference numbers: DZ1530, DZ1420, DZ1445, DZ1427). Video recordings of vocalizing males were collected for all four species using a Canon EOS 60D digital SLR Camera and a Sony DCR-SR45 camcorder using the night vision mode.

### Acoustical analyses

Only calls having a high signal to noise ratios that were free from overlapping calls of nearby males were used for the analysis. A total of 100 calls were measured from each species (20 calls per individual) except for one species, *M*. *zeylanica*, for which only 53 calls from three individuals were used due to the paucity of non-overlapping calls. Calls emitted by all species contained multiple pulses. Two species, *M*. *karunaratnei* and *M*. *zeylanica*, vary their call by dropping pulses (discontinuing the pulse train while maintaining longer duration between pulses several times within a call); however, this was observed only in five and four calls of the two species respectively. We excluded such calls from the analysis.

We measured eight call properties for this study (Table 1). These included temporal properties (call duration, call rise time, call fall time, 50% call rise time, 50% call fall time, number of pulses per call and pulse rate) and a spectral property (call dominant frequency by averaging spectrum over an entire call). Call characters were measured using methods and terminology from previous studies [1,19,20] as illustrated in S1 Fig. Raven Pro 1.4. was used to measure the call characters; temporal call characters were measured using Raven’s waveform display and spectral properties were measured by averaging the spectrum over the entire duration of a call (256 pt. fast fourier transform, Hanning window). Descriptive statistics of the call characters; mean ($\bar{\text{X}}$), standard deviation (SD), range and percent coefficient of variation ($\text{CV }=\text{ SD}/\bar{\text{X}}\;\times \;100$; calculated as within species CV, where SD is divided by the mean value of a species) were computed using Microsoft Excel 2010. Median and interquartile range were calculated for indivisible characters (number of pulses per call). Systat version 11 (SYSTAT 11) was used to conduct a principal component analysis (PCA) on the correlations matrix, using four call property variables. Call variables with high CV values (i.e. having CVs above 35%); Call rise time, Call fall time, 50% call rise time, 50% call fall time were excluded from the PCA analysis. We also did a discriminant function analysis (DFA) using six call variables (two highly correlated call variables to call duration, call rise time and 50% call rise time, were removed from the DFA analysis) and produced a canonical means plot using the first two canonical variables to verify the results of the PCA. For all four species, we assessed the relationship of the call variables with temperature, SVL and body weight using Pearson product moment correlation analyses (correlation coefficient = ρ).

**Table 1:** Descriptions of acoustic properties measured.

Properties of calls	
Call duration (ms)	Time between onset of first pulse and offset of last pulse in a call
Call rise time (ms)	Time between onset of first pulse and pulse of maximum amplitude
Call fall time (ms)	Time between pulse of maximum amplitude and offset of last pulse
50% Call rise time (ms)	Time between call onset and the half-amplitude point of earliest maximum peak in the call waveform
50% Call fall time (ms)	Time between the half-amplitude point of the last maximum peak in the call waveform and pulse offset
Pulses per call	Count of pulses (k)
Pulse rate (pulses/s)	(k– 1)/t, where t is the time between onset of first pulse and onset of last pulse
Dominant frequency (kHz)	Maximum frequency using Raven’s selection spectrum function over the duration of the entire call

## Results

Among the four species, *M*. *mihintalei* is the largest (mean SVL = 24.6 ± 2 mm, *N* = 5), and *M*. *karunaratnei* the smallest (SVL = 16.7 ± 0.9 mm, *N* = 5) with *M*. *ornata* (mean SVL = 19.2 ± 1.7 mm, *N* = 5) and *M*. *zeylanica* (mean SVL = 18.3 ± 1 mm, *N* = 3) were in between in body size. The body sizes of the two cool-adapted montane isolates were smaller than the two warm-adapted lowland dry zone forms; air temperature of the calling sites of *M*. *karunaratnei* and *M*. *zeylanica*, the two montane isolates, were 19.1°C and 18.2°C respectively; the temperatures at the two dry lowland habitat of *M*. *mihintalei* and *M*. *ornata* were 24.6°C and 25.2°C respectively. Temperature of the calling sites did not vary beyond ± 0.2°C across all recordings for each of the species. This was because, for a given species, the recordings were made at a single location within maximum of two consecutive nights. Results of the correlation analyses of all call variables against SVL, temperature and body weight for the four species show that temperature of the calling site negatively correlate with call duration (ρ = -0.86), call rise time (ρ = -0.88), 50% call rise time (ρ = -0.89) and pulses per call (ρ = -0.97), whereas SVL and body weight negatively correlate with dominant frequency (ρ = -0.73, ρ = -0.70) and pulses per call (ρ = -0.70, ρ = -0.78) respectively.

**Fig 2:**
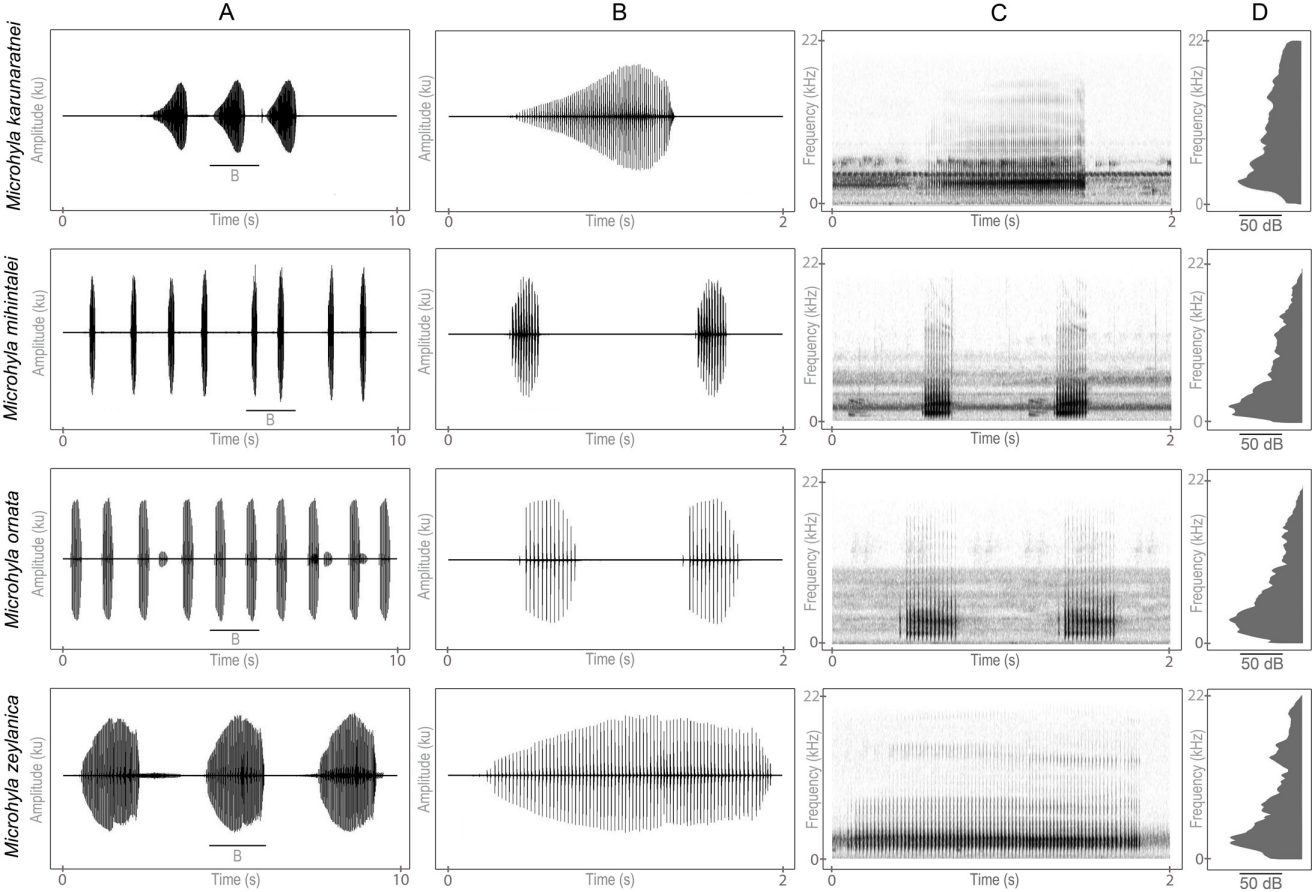
Advertisement calls of the four *Microhyla* species. (A) Ten second segment of a call by a single male. (B) Two second segment showing a single call underlined in A. (C) Spectrogram of the call shown in B. (D) Power spectrum of the call averaged over the duration of each call depicted in B (256 FFT size, Hanning window).

Hierarchical organizations of calls (i.e. call groups and call bouts) were not considered as they vary widely across the recordings. For these species, calls were emitted as duets or in groups. A single male always initiated calling following periodic pauses, which induced other males to call; this was observed for all four species. Waveform, spectrogram and power spectrum of the most common call type (hereafter referred to as advertisement call) of the four *Microhyla* species are illustrated in Fig 2. Pulsatile structure of the call, emitted as a short series and the presence of a single prominent frequency band is common to all species (Fig 2). Frequency modulations were not observed in any species.

The advertisement call of *M*. *karunaratnei* contains between 50 to 95 pulses having an average pulse rate of 77 pulses per second. The call duration ranged between 700 and 1172 ms. ($\bar{\text{X}}\;=\;866\;\pm \;101\text{ ms}$). Call dominant frequency ranged between 3.1 and 3.4 kHz ($\bar{\text{X}}\;=\;3.3\;\pm \;0.1\text{ kHz}$). It takes an average of 661 ms to reach its maximum amplitude and decreases rapidly within the next 205 ms. Call duration of *M*. *zeylanica* ranged between 1500 and 2000 ms ($\bar{\text{X}}\;=\;1852\;\pm \;123\text{ ms}$). It contains 61 to 92 pulses having an averaged pulse rate of 44 pulses per second. The call rises gradually within 1273 ± 243 ms and decreases slowly over the last 575 ± 178 ms. The advertisement call of *M*. *mihintalei* and *M*. *ornata* were short, less than half a second. Advertisement calls of *M*. *mihintalei* consist of 9 to 15 pulses, which had a rate of 58 pulses per second. Call duration ranged between 141 and 245 ms ($\bar{\text{X}}\;=\;187\;\pm \;24\text{ ms}$). Call typically reached its full amplitude under 144 ms ($\bar{\text{X}}\;=\;80\;\pm \;30\text{ ms}$) and decreased in amplitude over the last 106 ± 44 ms. Dominant frequency ranged between 1.3 and 2.6 kHz. Call duration of *M*. *ornata* was 300 ± 44 ms (210–795 ms). It composed 9 to 14 pulses, having a pulse rate of 41 pulses per second. Dominant frequency ranged between 2.2 and 3.4 kHz. Average call rise time and fall time were 154 ± 43 ms and 145 ± 61 ms respectively (Table 2).

**Table 2:** Descriptive statistics for calls of the four *Microhyla* species based on values determined from a sample of 100 calls from 5 males of each species (except *M*. *zeylanica*, 53 calls from 3 males).

	Mean	SD	Range (min–max)	CV%
***Microhyla karunaratnei***				
Call duration (ms)	866	101.7	699–1172	11.7
Call rise time (ms)	661	121.3	473–1009	18.3
Call fall time (ms)	205	40.6	112–306	19.8
50% Call rise time (ms)	353	113.5	199–763	32.1
50% Call fall time (ms)	44	13.6	16–92	31
Dominant frequency (kHz)	3.3	0.1	3.1–3.4	4.2
Pulses per call	66.5^a^	14.2^b^	50–95	14.4
Pulse rate	76.9	5.8	64.6–86.7	7.5
***Microhyla zeylanica***				
Call duration (ms)	1852	123	1503–1999	6.6
Call rise time (ms)	1273	243	784–1634	19
Call fall time (ms)	575	178	157–979	31
50% Call rise time (ms)	527	169	204–802	32
50% Call fall time (ms)	70	27	18–141	38.6
Dominant frequency (Hz)	2.6	0.2	2.2–2.9	10.2
Pulses per call	84^a^	5^b^	61–92	6.8
Pulse rate (pulses/s)	44.5	3.2	37–49	7.1
***Microhyla mihintalei***				
Call duration (ms)	187	24	141–245	12.8
Call rise time (ms)	80	29.9	44–144	36.8
Call fall time (ms)	106	44.2	35–199	41.4
50% Call rise time (ms)	27	11.2	14–53	40.6
50% Call fall time (ms)	26	22.7	3–79	86.4
Dominant frequency (kHz)	2.1	0.4	1.3–2.6	21.3
Pulses per call	13^a^	2.5^b^	9–15	10.3
Pulse rate (pulses/s)	58.6	2.8	49.8–66.1	14.0
***Microhyla ornata***				
Call duration (ms)	300	57	210–795	19
Call rise time (ms)	154	43	47–207	28
Call fall time (ms)	145	61	75–675	42.4
50% Call rise time (ms)	31	7	19–46	23.2
50% Call fall time (ms)	34	18	3–89	52.5
Dominant frequency (kHz)	3.1	0.4	2.2–3.4	13.4
Pulses per call	13^a^	1^b^	9–14	8.2
Pulse rate (pulses/s)	41.6	1.5	36.8–44.9	9.6

^a^ Median instead of mean

^b^ Interquartile range instead of SD.

Four temporal characters, call rise time, call fall time, 50% call rise time and 50% call fall time of the four species show the highest variation within species (CV range between 19% and 86.4%), whereas other four characters, call duration, dominant frequency, pulse rate and pulses per call were less variable, CV below 19% (Table 1).

**Fig 3:**
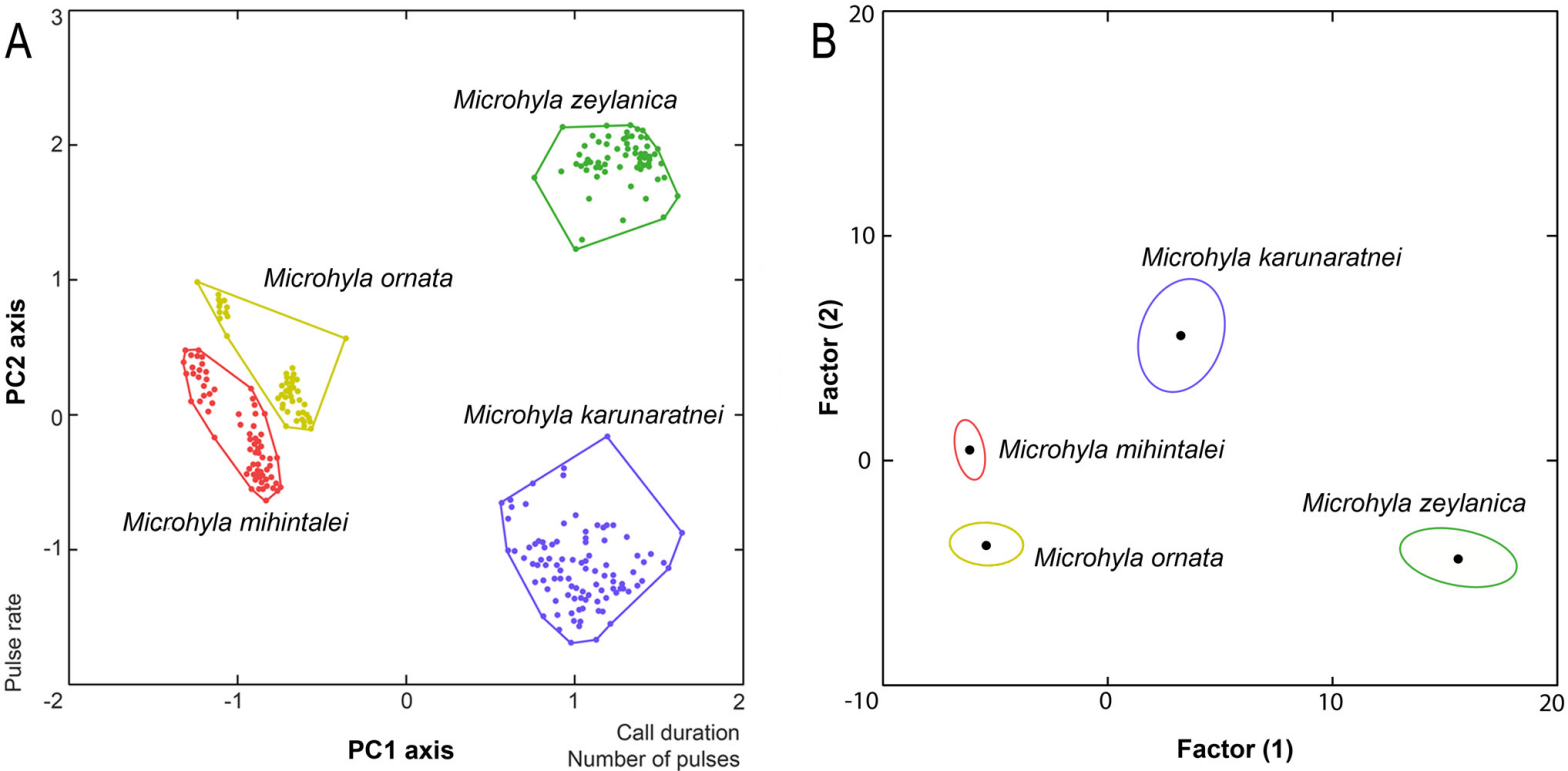
The principal component analysis and the discriminant function analysis of call characters for the four *Microhyla* species. (A) Plot of principle components 1 and 2 (PC1 vs. PC2) of the four call characters (call duration, dominant frequency, number of pulses per call and pulse rate). (B) Canonical variables plot of the discriminant function analysis of eight call character variables with 95% confidence ellipses and the centroids indicated.

The Principal Component analysis (Fig 3) on the correlations matrix, using four call property variables (Call duration, Dominant frequency, Number of pulses per call and Pulse rate) from the four species shows that none of the species overlap in PC space. PC 1 axis explains 53% of the total variation, mostly by the number of pulses per call (factor score = 0.985) and call duration (factor score = 0.874); PC 2 axis is explained by the pulse rate (factor score = -0.805), which explains 25% of the variance. *Microhyla zeylanica* overlaps with *M*. *karunaratnei* in PC1 axis, but does not overlap with any other form in PC2 axis. *Microhyla karunaratnei* slightly overlaps with both *M*. *ornata* and *M*. *mihintalei* in PC2 axis. Though *M*. *ornata* and *M*. *mihintalei* are separate on the PC space, they overlap in both axes. Further the stepwise backward DFA (Fig 3) identified each species more than 99% as correct (100% of *M*. *karunaratnei* and *M*. *zeylanica* and 99% of *M*. *ornata* and *M*. *mihintalei*, Wilks' lambda 0.0002); similar results were obtained for the jackknifed dataset. Three discriminant functions were generated, and eigenvalues of the first two variables were 59.09 and 15.76 respectively. The centroids are 3.27, 5.562 for *M*. *karunaratnei*, -6.132, 0.465 for *M*. *mihintalei*, -5.4, 3.746 for *M*. *ornata* and 15.588, -4.304 for *M*. *zeylanica*. The first canonical variable represents mostly call duration (canonical discriminant function, CDF, 14.116), 50% call fall time (CDF 4.356) and call fall time (CDF 3.325). The second canonical variable represents mostly 50% call fall time (CDF 6.478) and call fall time (CDF -2.386).

## Discussion

All four species studied here have pulsatile calls, which also seem to be a characteristic feature of the genus [26–33]. Hence the vocal repertoire within the genus is less complex when compared to other taxa studied from the region, such as most rhacophorids, ranids and bufonids [9,14,20,39,40]. Most of the call characters can be explained in relation to phylogenetic relatedness and by habitat occupation. For microhylids, the phylogenetic relatedness seems to have played a greater role than the habitat influence in the evolution of call characters [41].

Almost all members of the genus are pool breeders where the physical resources needed for females are concentrated. The males advertising in choruses from such optimal habitats are well known for *Microhyla* [26,29,31]. Choruses are also useful in several aspects such as increasing attraction to females and reducing predation risk by finding refuge in numbers [1]. But the cost of calling is higher in choruses due to individual reproductive success; hence the males make periodic pauses, which are essential to recover and conserve their energy.

A well-known strategy for adapting to increasing ambient temperature is shortening the call duration and reducing the number of pulses [42,43]. This is evident for the cool-adapted *M*. *karunaratnei* and *M*. *zeylanica* in having comparatively longer calls with higher number of pulses when compared to the members inhabiting the warmer drier lowlands.

Among the four Sri Lankan species, *M*. *mihintalei* shows the highest within species variation in all call characters except for call duration and call fall time. However wider comparisons cannot be done because most other studies have considered only a single individual.

Vocalization of *M*. *karunaratnei* and *M*. *zeylanica* were distinct from all others especially having longer call durations where the call duration of all other members were below 0.85 s (S1 Table). In fact *M*. *zeylanica* has the longest call duration (1.5 to 2 s) among all microhylids. Pulse rate of *M*. *rubra* from India has been reported as having the highest (108 pulses/s) among the clade (S1 Table). This may have occurred as a result of following a different terminology when characterizing the pulses. *Microhyla petrigena* is distinct within the group in having several unique characters such as the smallest in body size (SVL = 14 mm), highest dominant frequency (5100 Hz) and highest pulse rate (89 pulses/s). Call characters of *M*. *ornata* from India are similar to that of the Sri Lankan population. Vocalization (call characters) of *M*. *laterite* and *M*. *sholigari* are closer to the two Sri Lankan species *M*. *karunaratnei* and *M*. *zeylanica* than to other members of *Microhyla*. *Microhyla laterite*, so far, has the highest number of pulses per call among all congeners (S1 Table).

The only known locations of *M*. *karunaratnei* had been subjected to small-scale gem mining. These frogs however now are utilizing those abandoned gem pits, now overgrown with vegetation, for breeding. It shows that they are somewhat adaptable to changing conditions. Acoustic exploration of potential habitats can be used for rapid and accurate identification of new populations of this cryptic frog.

Acoustic analysis made here facilitates the comparative understanding of vocalization for the genus. Furthermore, this work will help non-destructive anuran surveys, identification of new populations, population monitoring and behavioral experimentation.

## Supporting Information

S1 Fig Illustration of the eight call characters measured. (JPG)

S1 Audio Sample recordings of the advertisement call of *Microhyla karunaratnei*. (ZIP)

S2 Audio Sample recordings of the advertisement call of *Microhyla zeylanica*. (ZIP)

S3 Audio Sample recordings of the advertisement call of *Microhyla mihintalei*. (ZIP)

S4 Audio Sample recordings of the advertisement call of *Microhyla ornate*. (ZIP)

S1 Table Summary of the common call characters of the members representing the genus, *Microhyla* that have been studied so far. (DOCX)

## References

[1] Gerhardt HC, Huber F. Acoustic communication in insects and anurans: common problems and diverse solutions: University of Chicago Press; 2002.

[2] Kelley DB. Vocal communication in frogs. Curr Opin Neurobiol. 2004; 14(6):751–757. 10.1016/j.conb.2004.10.015 15582379

[3] Narins PM, Feng AS, Fay RR, Popper AN, editors. Hearing and Sound Communication in Amphibians Springer Handbook of Auditory Research. Springer New York; 2006 10.1007/978-0-387-47796-1

[4] Wells KD. The ecology and behavior of amphibians: University of Chicago Press; 2010.

[5] Padial JM, De la Riva I. Integrative taxonomy reveals cryptic Amazonian species of Pristimantis (Anura: Strabomantidae). Zool J Linn Soc. 2009; 155(1):97–122. 10.1111/j.1096-3642.2008.00424.x

[6] Vieites DR, Wollenberg KC, Andreone F, Köhler J, Glaw F, Vences M. Vast underestimation of Madagascar's biodiversity evidenced by an integrative amphibian inventory. Proc Natl Acad Sci USA. 2009; 106(20):8267–72. 10.1073/pnas.0810821106 19416818 PMC2688882

[7] Angulo A, Icochea J. Cryptic species complexes, widespread species and conservation: lessons from Amazonian frogs of the Leptodactylus marmoratus group (Anura: Leptodactylidae). System Biodivers. 2010; 8(3):357–70. 10.1080/14772000.2010.507264

[8] Glaw F, Koehler J, De la Riva I, Vieites DR, Vences M. Integrative taxonomy of Malagasy tree frogs: combination of molecular genetics, bioacoustics and comparative morphology reveals twelve additional species of Boophis. Zootaxa. 2010; 2383(1):82.

[9] Meegaskumbura M, Senevirathne G, Wijayathilaka N, Jayawardena B, Bandara C, Manamendra-Arachchi K, et al The Sri Lankan torrent toads (Bufonidae: Adenominae: Adenomus): species boundaries assessed using multiple criteria. Zootaxa. 2015; 3911(1): 245–61. 10.11646/zootaxa.3911.2.6 25661609

[10] Wijayathilaka N, Garg S, Senevirathne G, Karunarathna N, Biju SD, Meegaskumbura M. A new species of Microhyla (Anura: Microhylidae) from Sri Lanka: an integrative taxonomic approach. Zootaxa. 2016; 4066(3): 331–342. 10.11646/zootaxa.4066.3.9 27395556

[11] Márquez R, Eekhout XR. Advertisement calls of six species of anurans from Bali, Republic of Indonesia. J. Nat. Hist. 2006; 40(9–10):571–88. 10.1080/00222930600712129

[12] Wells KD. The social behaviour of anuran amphibians. Anim Behav. 1977; 25: 666–93. 10.1016/0003-3472(77)90118-x

[13] Rand AS, Ryan MJ. The adaptive significance of a complex vocal repertoire in a neotropical frog. Z Tierpsychol. 1981; 57(34):209–14. 10.1111/j.1439-0310.1981.tb01923.x

[14] Arak A. Vocal interactions, call matching and territoriality in a Sri Lankan tree frog, Philautus leucorhinus (Rhacophoridae). Anim Behav. 1983; 31(1):292–302. 10.1016/s0003-3472(83)80199-7

[15] Wells KD, Schwartz JJ. The behavioral ecology of anuran communication In: Hearing and sound communication in amphibians: Springer; 2007 pp. 44–86. 10.1007/978-0-387-47796-1_3

[16] Bridges AS, Dorcas ME. Temporal variation in anuran calling behavior: implications for surveys and monitoring programs. Copeia. 2000; 2000(2):587–592. 10.1643/0045-8511(2000)000[0587:tviacb]2.0.co;2

[17] Aide TM, Corrada-Bravo C, Campos-Cerqueira M, Milan C, Vega G, Alvarez R. Real-time bioacoustics monitoring and automated species identification PeerJ. 2013; 1:e103 10.7717/peerj.103 23882441 PMC3719130

[18] Heyer R, Donnelly MA, Foster M, Mcdiarmid R, editors. Measuring and monitoring biological diversity: standard methods for amphibians. Smithsonian Institution; 2014.

[19] Bee MA, Suyesh R, Biju SD. The vocal repertoire of Pseudophilautus kani, a shrub frog (Anura: Rhacophoridae) from the Western Ghats of India. Bioacoustics. 2013; 1;22(1):67–85. 10.1080/09524622.2012.712750

[20] Bee MA, Suyesh R, Biju SD. Vocal behavior of the Ponmudi bush frog (Raorchestes graminirupes): repertoire and individual variation. Herpetologica. 2013; 69(1):22–35. 10.1655/herpetologica-d-11-00042

[21] Frost DR. Amphibian Species of the World: an Online Reference. 2015; Version 6.0 Available: http://research.amnh.org/herpetology/amphibia/index.html. American Museum of Natural History, New York, USA Accessed 2 November 2015.

[22] Meegaskumbura M, Bossuyt F, Pethiyagoda R, Manamendra-Arachchi K, Bahir M, Milinkovitch MC, et al Sri Lanka: an amphibian hot spot. Science. 2002; 298(5592):379 10.1126/science.298.5592.379 12376694

[23] Bossuyt F, Meegaskumbura M, Beenaerts N, Gower DJ, Pethiyagoda R, Roelants K, et al Local endemism within the Western Ghats-Sri Lanka biodiversity hotspot. Science. 2004; 306(5695):479–81. 10.1126/science.1100167 15486298

[24] The IUCN Red List of Threatened Species. 2015; Version 2015–4. Available: www.iucnredlist.org. Accessed 04 January 2016.

[25] The Zoological Society of London, The EDGE of Existence programme. 2015; Available: http://www.edgeofexistence.org/amphibians/top_100.php. Accessed 26 November 2015.

[26] Dring JCM. Amphibians and reptiles from northern Trengganu, Malaysia, with descriptions of two new geckos: Cnemaspis and Cyrtodactylus. Bull Br Mus. 1979; 34.

[27] Heyer WR. Mating calls of some frogs from Thailand: Field Museum of Natural History; 1971 10.5962/bhl.title.2934

[28] Kuramoto M. Advertisement Calls of Two Taiwan Microhylid Frogs, Microhyla heymonsi and M. ornata: Taxonomy. Zoolog Sci. 1987; 4(3):563–7.

[29] Kanamadi R, Hiremath C, Schneider H. Courtship, amplexus and advertisement call of the frog, *Microhyla rubra*. Curr Sci. 1994; 66(9):683–4.

[30] Kuramoto M, Joshy SH. Morphological and acoustic comparisons of Microhyla ornata, M. fissipes, and M. okinavensis (Anura: Microhylidae). Curr Herpetol. 2006; 25(1):15–27.

[31] Dehling JM. Advertisement calls of two species of Microhyla (Anura: Microhylidae) from Borneo. Salamandra. 2010; 46(2):114–116.

[32] Grosselet O, Sengupta S, Gupta A, Vauche M, Gupta S. Microhyla heymonsi Vogt, 1911 (Anura: Microhylidae) from mainland India, with bioacoustic analysis of its advertising call. Hamadryad. 2004; 29(1):131–133.

[33] Seshadri KS, Singal R, Priti H, Ravikanth G, Vidisha MK, Saurabh S, et al Microhyla laterite sp. nov., A New Species of Microhyla Tschudi, 1838 (Amphibia: Anura: Microhylidae) from a Laterite Rock Formation in South West India. PloS one. 2016; 9–11(3). 10.1371/journal.pone.0149727 PMC4784882 26960208

[34] Toledo LF, Tipp C, Márquez R. The value of audiovisual archives. Science. 2015; 347 (6221): 484 10.1126/science.347.6221.484-b 25635077

[35] Fernando P, Siriwardhane M. Microhyla karunaratnei (Anura: Microhylidae), a new species of frog endemic to Sri Lanka. J. South Asian Nat Hist. 1996; 2(1):135–42.

[36] Howlader MS, Nair A, Gopalan SV, Merilä J. A New Species of Microhyla (Anura: Microhylidae) from Nilphamari, Bangladesh. PloS one. 2015;10(3) 10.1371/journal.pone.0119825 PMC4373918 25806804

[37] Parker HW, Osman-Hill WC. Frogs of the genus Microhyla from Ceylon. Annals and Magazine of Natural History (Series 12). 1949; 1: 759–764.

[38] Dutta SK, Manamendra-Arachchi K. The Amphibian Fauna of Sri Lanka. Colombo, Sri Lanka: Wildlife Heritage Trust of Sri Lanka; 1996.

[39] Bee MA, Kozich CE, Blackwell KJ, Gerhardt HC. Individual variation in advertisement calls of territorial male green frogs, Rana clamitans: implications for individual discrimination. Ethology. 2001; 107(1):65–84. 10.1046/j.1439-0310.2001.00640.x

[40] Cocroft RB, Ryan MJ. Patterns of advertisement call evolution in toads and chorus frogs. Anim Behav. 1995; 49(2): 283–303. 10.1006/anbe.1995.0043

[41] Bosch J, De la Riva I. Are frog calls modulated by the environment? An analysis with anuran species from Bolivia. Can J Zool. 2004; 82(6):880–.

[42] Gayou DC. Effects of temperature on the mating call of *Hyla versicolor*. Copeia. 1984:733–8. 10.2307/1445157

[43] Narins PM, Meenderink SW. Climate change and frog calls: long-term correlations along a tropical altitudinal gradient. Proc Biol Sci. 2014; 281(1783). 10.1098/rspb.2014.0401 PMC3996621 24718765

