# Supplementary figures and images for: An Acoustic Analysis of the Genus Microhyla (Anura: Microhylidae) of Sri Lanka
Source: PLoS One. 2016 Jul 12;11(7):e0159003. doi: 10.1371/journal.pone.0159003 (PMC4942061; doi:10.1371/journal.pone.0159003)

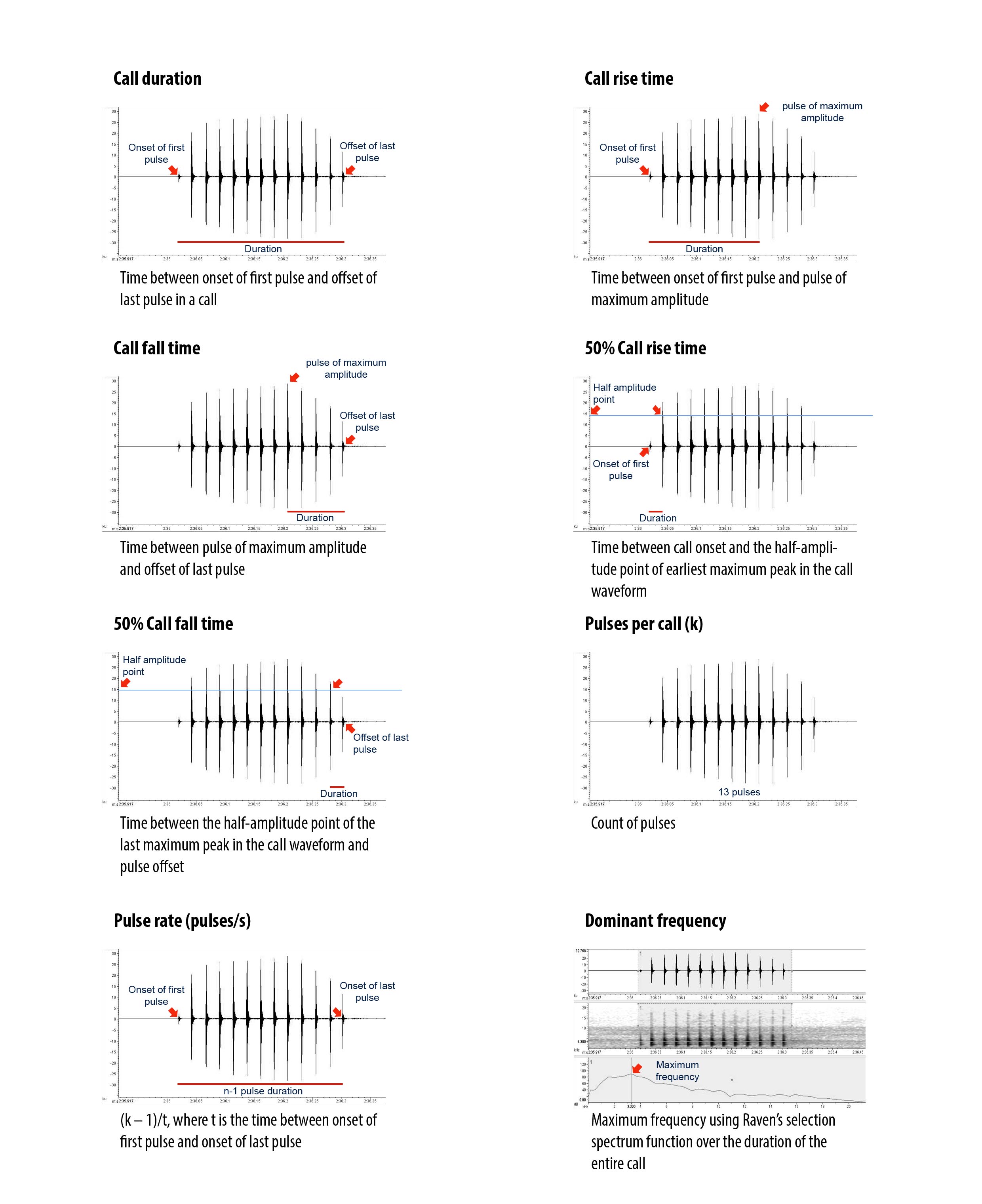

Supplement: S1 Fig — (JPG) [file pone.0159003.s001.jpg]
