# Supplementary material for: An Acoustic Analysis of the Genus Microhyla (Anura: Microhylidae) of Sri Lanka
Source: PLoS One. 2016 Jul 12;11(7):e0159003. doi: 10.1371/journal.pone.0159003 (PMC4942061; doi:10.1371/journal.pone.0159003)
Supplement: S1 Table — (DOCX) [file pone.0159003.s006.docx]

| **Species** | **Number of calls (males) analyzed** | **Number of pulses per call (min-max)** | **Call duration (ms)** | **Pulse rate (number of pulses per second)** | **Dominant frequency range (Hz)** | **SVL (mm)** | **Location** | **Temperature (ºC)** | **Ref.** |
| --- | --- | --- | --- | --- | --- | --- | --- | --- | --- |
| *Microhyla berdmorei* | 3 (1) | 3-9 | 90-260 | 33-35 | 1500-1800 | N | Thailand | N | Heyer, 1971 |
| *Microhyla borneensis* | 76 | 4-8 | 169 | 30 | 1400-2900 | 17-21 | Borneo | 23.5 | Dehling, 2010 |
| *Microhyla butleri* | 11 (2) | 6-7 | 160-210 | 28-44 | 1200-4500 | N | Thailand | N | Heyer, 1971 |
| *Microhyla fissipes* | 15 (3) | 10-18 | 230-310 | 53-60 | 1200-3500 | N | Thailand | 25-28 | Heyer, 1971 |
| *Microhyla heymonsi* | 1 (1) | 11 | 480 | 23 | 1700-3000 | N | Thailand | 28 | Heyer, 1971 |
| *Microhyla heymonsi* | N (1) | 17 (5)* | 171 (33)* | 26-39 | 1300-3700 | 24.4 | India | N | Grosselet *et al,* 2004 |
| *Microhyla laterite* | 2 (8) | 90-126 | 600-850 | N | 3538-3664 | N | India | 27.9 | Seshadri *et al*, 2016 |
| *Microhyla petrigena* | (6) | 6-17 | 133 | 89 | 3800-5100 | 14-16 | Borneo | 24.1 | Dehling, 2010 |
| *Microhyla ornata* | N | 8-13 | 280 (19)* | 35.9 (1.08)* | 1000-4000 | 22-25 | India | 25.5 | Kuramoto & Joshy, 2006 |
| *Microhyla ornata* | 100 (5) | 9-14 | 210-332 | 37-45 | 2200-3400 | 18-21 | Sri Lanka | 25.2 | In this manuscript |
| *Microhyla rubra* | (10) | 15-21 | 138- 228 | 108 | 2268 (43)* | N | India | N | Kanamadi *et al*, 1994 |
| *Microhyla sholigari* | N (8) | 64-72 | 530-810 | N | 3518-3779 | N | India | 23.2 | Seshadri *et al*, 2016 |
| *Microhyla mihintalei* | 100 (5) | 9-15 | 141-245 | 50-66 | 1300-2600 | 22-27 | Sri Lanka | 24.6 | In this manuscript |
| *Microhyla karunaratnei* | 100 (5) | 50-95 | 699-1172 | 65-87 | 3100-3400 | 16-18 | Sri Lanka | 19.1 | In this manuscript |
| *Microhyla zeylanica* | 53 (3) | 61-92 | 1503-1999 | 37-49 | 2200-2900 | 17-19 | Sri Lanka | 18.2 | In this manuscript |

**S1 Table. Summary of the common call characters of the members representing the genus *Microhyla* that have been studied so far.**

* Mean value was given instead of range, standard deviation is within brackets. N- not given

References:

1. Heyer WR. Mating calls of some frogs from Thailand: Field Museum of Natural History; 1971.
2. Dehling JM. Advertisement calls of two species of *Microhyla* (Anura: Microhylidae) from Borneo. Salamandra. 2010; 46(2):114−116.
3. Grosselet O, Sengupta S, Gupta A, Vauche M, Gupta S. *Microhyla heymonsi* Vogt, 1911 (Anura: Microhylidae) from mainland India, with bioacoustic analysis of its advertising call. Hamadryad. 2004; 29(1):131−133.
4. Seshadri KS, Singal R, Priti H, Ravikanth G, Vidisha MK, Saurabh S, Pratik M, Gururaja KV. *Microhyla laterite* sp. nov., A New Species of *Microhyla* Tschudi, 1838 (Amphibia: Anura: Microhylidae) from a Laterite Rock Formation in South West India. PloS one. 2016; 9-11(3).
5. Kuramoto M, Joshy SH. Morphological and acoustic comparisons of *Microhyla ornata, M. fissipes*, and *M. okinavensis* (Anura: Microhylidae). Curr Herpetol. 2006; 25(1):15−27.
6. Kanamadi R, Hiremath C, Schneider H. Courtship, amplexus and advertisement call of the frog, *Microhyla rubra*. Curr Sci. 1994; 66(9):683−4.
